# Sex-Dependent Pathology in the HPA Axis at a Sub-acute Period After Experimental Traumatic Brain Injury

**DOI:** 10.3389/fneur.2020.00946

**Published:** 2020-09-30

**Authors:** Caitlin E. Bromberg, Andrew M. Condon, Samantha W. Ridgway, Gokul Krishna, Pamela C. Garcia-Filion, P. David Adelson, Rachel K. Rowe, Theresa Currier Thomas

**Affiliations:** ^1^Barrow Neurological Institute at Phoenix Children's Hospital, Phoenix, AZ, United States; ^2^Department of Child Health, University of Arizona College of Medicine-Phoenix, Phoenix, AZ, United States; ^3^Department of Biology and Biochemistry, University of Bath, Bath, United Kingdom; ^4^Department of Biology, School of Life Sciences, Arizona State University, Tempe, AZ, United States; ^5^Department of Biomedical Informatics, University of Arizona College of Medicine-Phoenix, Phoenix, AZ, United States; ^6^Department of Neurosurgery, Mayo Clinic School of Medicine, Phoenix, AZ, United States; ^7^School of Biological and Health Systems Engineering, Arizona State University, Tempe, AZ, United States; ^8^Phoenix VA Health Care System, Phoenix, AZ, United States

**Keywords:** hypothalamic-pituitary-adrenal axis, diffuse traumatic brain injury, sex-differences, glucocorticoid receptors, neuroinflammation, astrocytosis, microglia, diffuse axonal injury

## Abstract

Over 2.8 million traumatic brain injuries (TBIs) are reported in the United States annually, of which, over 75% are mild TBIs with diffuse axonal injury (DAI) as the primary pathology. TBI instigates a stress response that stimulates the hypothalamic-pituitary-adrenal (HPA) axis concurrently with DAI in brain regions responsible for feedback regulation. While the incidence of affective symptoms is high in both men and women, presentation is more prevalent and severe in women. Few studies have longitudinally evaluated the etiology underlying late-onset affective symptoms after mild TBI and even fewer have included females in the experimental design. In the experimental TBI model employed in this study, evidence of chronic HPA dysregulation has been reported at 2 months post-injury in male rats, with peak neuropathology in other regions of the brain at 7 days post-injury (DPI). We predicted that mechanisms leading to dysregulation of the HPA axis in male and female rats would be most evident at 7 DPI, the sub-acute time point. Young adult age-matched male and naturally cycling female Sprague Dawley rats were subjected to midline fluid percussion injury (mFPI) or sham surgery. Corticotropin releasing hormone, gliosis, and glucocorticoid receptor (GR) levels were evaluated in the hypothalamus and hippocampus, along with baseline plasma adrenocorticotropic hormone (ACTH) and adrenal gland weights. Microglial response in the paraventricular nucleus of the hypothalamus indicated mild neuroinflammation in males compared to sex-matched shams, but not females. Evidence of microglia activation in the dentate gyrus of the hippocampus was robust in both sexes compared with uninjured shams and there was evidence of a significant interaction between sex and injury regarding microglial cell count. GFAP intensity and astrocyte numbers increased as a function of injury, indicative of astrocytosis. GR protein levels were elevated 30% in the hippocampus of females in comparison to sex-matched shams. These data indicate sex-differences in sub-acute pathophysiology following DAI that precede late-onset HPA axis dysregulation. Further understanding of the etiology leading up to late-onset HPA axis dysregulation following DAI could identify targets to stabilize feedback, attenuate symptoms, and improve efficacy of rehabilitation and overall recovery.

## Highlights

- The etiology leading up to late-onset affective symptoms after mild TBI is unknown
- A single diffuse traumatic brain injury leads to sex-specific changes in the HPA axis
- Both injury-induced neuroinflammation and astrocytosis are greater in males compared with females
- TBI leads to increased GR protein levels in the hippocampus of females, but not males

## Introduction

There are 2.87 million reported traumatic brain injuries (TBIs) every year in the United States. Of these, over 75% include diffuse axonal injury (DAI) (1, 2). Yet, the true incidence of TBI is unknown as many do not seek medical care. According to the National Women's Health Network, it is estimated that overall 20 million women have sustained at least one TBI from domestic violence in the United States—exceeding the numbers for athletes and military combined (3). Females are also more vulnerable to DAI due to lesser neck strength than their male counterparts, which leads to increased velocity of acceleration-deceleration forces (4). The World Health Organization predicts that TBI will become the third leading cause of death and disability in the world within the next year (5, 6). As a result, TBI has been carrying the moniker of a “silent epidemic.”

Primary damage from most forms of TBI, including DAI, can also include cell death, neuron process shearing, subarachnoid and petechial hemorrhage, subdural hematoma, and blood-brain barrier breakdown (7–9). DAI causes disrupted circuits that impact behaviorally relevant processing (10, 11). In addition to DAI, TBI also leads to secondary injury cascades and activated microglia and astrocytes that have been implicated in the pathophysiology contributing to late-onset post-TBI symptoms (12, 13). Neuroinflammatory responses are part of the secondary injury cascade that can be both beneficial and detrimental. Clinical studies reported a bidirectional association between neuroinflammatory load and circulating cortisol levels on 6-month outcome in severe TBI patients, indicative of neuroendocrine-immune dysfunction, where either an immune response to TBI was inadequate or neuroinflammation was prolonged (14). Microglia can promote neurotoxic, neuroprotective, and neuroplasticity events as well as clear damaged tissue (15). Astrocytes also play many roles in response to injury and throughout the repair process. Astrocytes can become hyperactive and form glial scars to mechanical injury, or promote survival via neuroprotective, immunomodulatory, antioxidant, angiogenic, and neuroplasticity roles (16). Both glial cells play pivotal roles in recovery, compensation, and successful rehabilitation after TBI (11, 17–20).

Post-TBI symptoms are persisting or late-onset and can linger for months and years post-injury, when stress disorders and other hypothalamic-pituitary-adrenal (HPA) axis related symptoms are prevalent (21). These symptoms can include: anxiety, depression, post-traumatic stress disorder, epilepsy, memory problems, mood swings, impulsivity, apathy, and sleep disorders (22–26). While reported in both men and women, women of child-bearing age report a higher incidence and severity of stress-related symptoms after mild TBI (27–31). Further, menses resumed when cortisol levels normalized after TBI-induced amenorrhea, indicating a connection between the hypothalamic-pituitary-gonadal (HPG) and HPA axes (32). Stress disorders during recovery from TBI can decrease adaptive plasticity and further compromise cognitive abilities, impair social interactions, motivation, and immunity (33, 34). These symptoms increase the likelihood of non-compliance with rehabilitation, decrease the benefit when compliant, and worsen long-term outcome (35).

TBI-induced damage to, or dysregulation of, the HPA axis is associated with increased rates of affective disorders (up to 50% of survivors) and endocrinopathies (between 30 and 50% of survivors) (36). In a recent UPFRONT study (prospective follow up study on mild TBI), 57% of mild TBI patients that were asymptomatic at 2-weeks post-injury became symptomatic over the following 12 month period, with a prevalence of late-onset affective symptoms (37). There is evidence that both clinical and experimental TBI can cause a surge in cortisol (corticosterone in rats; CORT) followed by a drawn-out decrease that results in chronic HPA axis dysregulation indicative of “adrenal fatigue” or a mild form of adrenal insufficiency (38). Chronic stress paradigms also initiate processes that lead toward this “mild” adrenal insufficiency, where neuroinflammation is noted in the hypothalamus which is likely mediated by glucocorticoid receptor (GR) activation on microglia (39–41). Our lab has previously reported lower resting CORT levels and a blunted response to restraint stress at 54 days post-injury (DPI) in brain injured male rats compared with uninjured shams (42). We have also reported a small but significant change in the complexity of paraventricular neurons of brain injured rats compared with shams without evidence of changes in overt neuropathology over time (42). CORT can also modulate signaling of several neurotransmitters through changes in levels and localization of mineralocorticoid (MR) and GR receptors, with the potential for a global role in the late-onset of affective and cognitive symptoms following mild TBI (37, 43–48). The purpose of these studies is to assess sub-acute changes to gliosis, GRs, and HPA axis regulation for indications of secondary pathological processes that can mediate the changes in HPA axis regulation at 54 DPI.

The HPA axis is a complex system involving several positive and negative feedback loops, brain regions, neurochemicals, and peripheral targets. Using the well-characterized midline fluid percussion injury (mFPI) model, these studies were designed to evaluate the influence of TBI on corticotropin-releasing hormone (CRH) expression, circulating ACTH levels, adrenal weights, the glial inflammatory response, and GR levels in the HPA axis of male and cycling female rats. The paraventricular nucleus (PVN) of the hypothalamus was the primary target of these studies. Since previous studies indicated very little neuropathology in the PVN and amygdala at 7 DPI, the hippocampus was identified as a critical relay for negative feedback to the HPA axis that is vulnerable to TBI-induced neuropathology (49). Specifically, the dentate gyrus (DG) is vulnerable to midline fluid percussion injury, demonstrates persisting neuropathology, and has the highest concentration of GRs in comparison to the rest of the hippocampus, suggesting a more integral role in the regulation of the HPA axis (50, 51).

## Materials and Methods

### Animals

A total of 44 young adult age-matched male and naturally cycling female Sprague-Dawley rats (males 367 g ± 3, females 235 g ± 1.5; age 3–4 months) (Envigo, Indianapolis, IN) were used in these experiments. For histology we used a subset of 20 rat brain hemispheres (*n* = 5/group; the other hemisphere was biopsied for gene/protein extraction). The remaining 24 rats were included to ensure 90% power to detect a 20% change from shams, with a total of 8–11 rats/group. Upon arrival, rats were given a 1-week acclimation period, housed in normal 12-h light-dark cycle (Red light: 18:00–06:00) and allowed access to food and water *ad libitum* (Teklad 2918, Envigo, Indianapolis, IN). Rats were pair housed according to injury status (i.e., injured housed with injured) and according to sex throughout the duration of the study. All procedures and animal care were conducted in compliance with an approved Institutional Animal Care and Use Committee protocol (18–384) at the University of Arizona College of Medicine-Phoenix which is consistent with the National Institutes of Health (NIH) Guidelines for the Care and Use of Laboratory Animals.

### Surgical Procedure

Midline fluid percussion injury (mFPI) surgery was carried out similarly to previously published methods from this laboratory (52, 53). Each cage of rats (2/cage) was randomized into either injured or sham groups following acclimation to the vivarium facility. Briefly, rats were anesthetized with 5% isoflurane in 100% O_2_, heads were shaved, rats were weighed, and placed into a stereotaxic frame (Kopf Instruments, Tujunga, CA) with a nosecone that maintained 2.5% isoflurane for the duration of the procedure. A 4.8 mm circular craniotomy was centered on the sagittal suture midway between bregma and lambda carefully ensuring that the underlying dura and superior sagittal sinus remained intact. An injury hub created from the female portion of a 20-gauge Luer-Lock needle hub was cut, beveled and placed directly above and in-line with the craniectomy site. A stainless-steel anchoring screw was then placed into a 1 mm hand-drilled hole into the right frontal bone. The injury hub was affixed over the craniectomy using cyanoacrylate gel and methyl-methacrylate (Hygenic Corp., Akron, OH) and filled with 0.9% sterile saline. The incision was then partially sutured closed on the anterior and posterior edges with 4.0 Ethilon sutures and topical lidocaine and antibiotic ointment were applied. Rats were returned to a warmed holding cage and monitored until ambulatory (~60–90 min).

### Injury Induction

Approximately 2 h following surgical procedures and the return of ambulation, rats were re-anesthetized using 5% isoflurane in 100% oxygen for 3 min. The injury hub was filled with 0.9% sterile saline and attached to the male end of a fluid percussion device (Custom design and Fabrication, Virginia Commonwealth University, Richmond, VA). After the return of a pedal withdrawal response, an injury averaging 1.8–2.0 atmospheric pressure (atm) for males and 1.7–1.9 atm for females was administered by releasing the pendulum (a 16° angle for males and 15.5° angle for female) onto the fluid-filled cylinder. Shams were attached to the fluid percussion device, but the pendulum was not released after a positive pedal withdrawal response. Immediately after administration of the injury, the forearm fencing response was recorded for injured animals and the injury hub was removed *en bloc*. Injured rats were monitored for the presence of apnea, seizures, and the return of righting reflex (54, 55). The righting reflex time is the total time from initial impact until the rat spontaneously rights itself from a supine position to a prone position. Inclusion criteria required that injured rats have a righting reflex time ranging from 6 to 10 min (males 7:56 avg., females 6:22) and a fencing response. Rats were re-anesthetized for 2 min to inspect the injury site for hematoma, herniation, and dural integrity. The injury site was then stapled closed (BD AutoClip^TM^, 9 mm) and topical lidocaine and antibiotic ointment were applied. Rats were then placed in a clean, warmed holding cage and monitored for at least 1 h following injury or sham surgery before being placed in a new, clean cage with bedding and returned to the vivarium housing room, where post-operative evaluations continued for 4 days post-injury. These cages were not changed for the duration of the 7 days to minimize stress and avoid confounds between cohorts. Post-operative monitoring included appearance of incision, monitoring of behavior, body weight measurements, and a pain scale evaluation. Rats were euthanized (and therefore excluded) if they lost more than 15% of their body weight or presented with chronic pain symptoms as described by the American Association for Accreditation of Laboratory Animal Care (AAALAC). No animals were excluded based on these conditions.

### Translational Relevance

mFPI has typically been carried out in Sprague-Dawley rats for over 30 years, with reproducible pathophysiology, neurochemical, and behavior outcomes relevant to clinical observation (54, 56). mFPI best models closed head injury with decompressive craniectomy by reproducing DAI without contusion or cavitation encompassing the hallmark pathology of clinical diffuse TBI. Autonomic dysfunction with hypothalamic origins has been identified in FPI models and TBI patients (57, 58). After regaining the righting reflex, injured rats require little to no medical intervention in the post-operative period, similar to mild TBI as defined by a Glasgow Coma Score of 13–15. More detailed discussion of the clinical relevance of mFPI have been published (53, 59). At 3–4 months of age, rats are roughly estimated to translationally represent late adolescent-young adult humans (60). This calculation is based on comparisons between weaning, sexual maturity, social maturity, menopause (females), and lifespan.

### Tissue Collection

At 7 DPI, tissue was collected between 07:00 and 11:00, to control for the influence of circadian rhythm on the HPA axis. A timer carried by the investigator retrieving each cage of rats was started as soon as they entered the home cage room. One cage of rats was quickly retrieved from their home room and brought to the necropsy suite where both rats were immediately placed in the induction chamber that had been pre-filled with isoflurane (~30–45 s) (5% isoflurane at 2.5 O_2_ rate). Rats were under full anesthesia within 190–220 s of being disturbed in their home cage, as observed with full loss of righting reflex, similar to previous reports (61). Rats were kept under anesthesia for 2 min total. Two teams were available (one for each rat), where rats were immediately removed from the induction chamber, weighed and decapitated. Trunk blood was collected into weigh boats precoated with ethylenediaminetetraacetic acid (EDTA), transferred to BD Microtainer™ MAP Microtubes (CAT#22-253-270 ThermoFisher), immediately centrifuged at 1,500 revolutions per minute (RPM) at 4°C for 10 min, and plasma siphoned off and stored for later use in a−80°C freezer. From the time the rats were disturbed in the housing room to decapitation ranged between 3 and 4 min.

Brains were extracted, rinsed with ice cold PBS, placed into a rat brain matrix, and cut into 2 mm coronal sections. The hypothalamus (~bregma −0.5 to −2.5 mm) and dorsal hippocampus (~bregma −2.5 to −4.5 mm) were biopsied (see Supplementary Figures 1, 2) and/or one hemisphere of each rat was collected for histology (Neuroscience Associates, Knoxville, TN) (62). Tissue biopsies were flash frozen and stored in a −80°C freezer until RNA/protein extraction. Adrenal glands were also collected from each rat. Excess fascia and fatty tissue were removed from the adrenal glands and weights were recorded. Both adrenal glands were weighed, and an average weight was calculated for each rat. The weights were normalized to the rat's body weight to calculate an individual organ index (adrenal gland weight/body weight = organ index).

### Tissue RNA/Protein Isolation

Samples were taken from −80°C and both RNA and protein were extracted using optimized protocols for the Qiagen AllPrep RNA/DNA/Protein mini Kit 50 (CAT No.: 80204, Qiagen Hilden, Germany). Biopsies were homogenized in 600 mL of a 1:100 ratio BME: Buffer RLT ratio using a FisherBrand Pellet Pestle mixer for 3 min. Lysate was centrifuged at 4°C for 3 min at full speed before the supernatant was transferred to an AllPrep DNA spin column and centrifuged again for 30 s at 10,000 RMP. The flow-through was combined with 100% ethanol and mixed well. The new mixture was transferred into a RNeasy spin column and centrifuged for 20 s at 10,000 RPM. The flow-through was poured into a new 2 mL tube and placed on ice for protein purification. Several buffer washes were added to the RNeasy spin column: Buffer RW1, Buffer RPE, and 80% ethanol. The RNeasy spin column was then centrifuged at full speed for 5 min to dry before adding 30 μl of RNase-free water and centrifuging again at 10,000 RPM for 1 min to elute the RNA. Once RNA was extracted it was measured using a NanoDrop (Thermo Fisher, Waltham MA) for concentration of RNA and 260:280 ratios. Inclusion criteria required that all samples have an RNA concentration >25 ng/μl or a 260/280 ratio between 1.9 and 2.1. A total of 4 males and 3 females in the hypothalamus and 1 male and 3 females in the hippocampus were excluded due to not meeting these criteria.

For protein, buffer APP, provided in the Qiagen kit, was added to the “protein” flow-through, mixed vigorously, and incubated at room temperature for 10 min before being centrifuged for 10 min at full speed. The supernatant was decanted and 500 μl of 70% ethanol was added. The tubes were centrifuged for 1 min at full speed, then all liquid was decanted, and the protein pellet was left to dry for at least 10 min. The protein pellet was resuspended in 5% sodium dodecyl sulfate (SDS) and incubated for 5 min at 95°C to completely dissolve and denature the protein. Protein concentrations were determined using the Bicinchoninic acid assay (BCA) following manufacturer's instructions (Pierce, Rockford, IL). Protein was divided into 10 μl aliquots and stored at −80°C. MicroPlate BCA Assay Kit from Thermo Fisher (cat no: 23252, Thermo Fisher Scientific, Waltham, MA). Inclusion criterion required protein concentrations be >0.5 μg/μl. Protein concentrations on average were at 6.0 μg/μl.

### Quantitative RT qPCR

Total RNA was reverse transcribed to cDNA using the High Capacity RNA-to-cDNA Kit from Life Technologies™ (catalog # 4387406), then diluted to 5 ng for RT qPCR using TaqMan® Gene Expression Assays for GR (Rn00561369_m1) and CRH (Rn00578611_m1). Assays were run in multiplex along with a biological control of Eukaryotic 18S ribosomal RNA (rRNA) (4310893E) and the TaqMan® Fast Advanced Master Mix (catalog # 4444963) in a ratio of 9 μl of master mix: 1 μl of gene: 1 μl 18S rRNA per well. Samples were run in triplicate. TaqMan® Fast Advanced Master Mix thermocycling protocols were used. Eukaryotic 18S rRNA was used as a biological control. For relative gene expression analysis, each sample was normalized to the 18S rRNA biological control and then to gene expression levels in the sham group using the 2^−ΔΔCT^ method (63).

### Automated Capillary Western

Protein levels were evaluated using automated capillary western (ProteinSimple®, San Jose, CA). Prior to running all samples, each protein of interest was optimized for primary antibody (see Supplementary Figure 3), antibody concentration, protein concentrations, multiplexing with housekeeping protein (glyceraldehyde 3-phosphate dehydrogenase; GAPDH), denaturing process, loading conditions, and exposure times. Secondary antibodies, streptavidin horseradish peroxidase (HRP), dithiothreitol (DTT), molecular weight fluorescent standards (loading control), luminol, peroxide, sample buffer, antibody diluent, running buffer, wash buffer, capillaries and plates (plates containing stacking matrix, separation matrix, and matrix removal buffer were purchased from ProteinSimple® and used according to manufacturer's recommendations).

After protein extractions, samples were prepared according to optimized conditions. Samples were combined with 1× sample buffer and master-mix (40 mM DTT, 10× sample buffer and 1× Fluorescent Standards) to achieve desired concentration and were denatured at 37°C for 30 min. The primary antibody was diluted with manufacturer's antibody diluent to desired concentration. The secondary antibody was combined with a 20× Anti-Rabbit HRP Conjugate (catalog# 043-4226, ProteinSimple®) so both the primary and housekeeping gene (GAPDH) could be multiplexed into the same capillary well. The ladder, samples, antibody diluent, diluted primary and secondary antibodies, streptavidin HRP, wash buffer, and chemiluminescent (luminol and peroxide at a 50:50 ratio) were then placed in the designated wells per experimental design. Each plate was centrifuged at 2,500 RPM for 5 min and placed into the automated capillary western machine, where proteins were separated by size (electrophoresis), immobilized, and immunoprobed in each capillary via a one-time use capillary cartridge. Conditions for running plates were not modified from manufacturer's settings. Every capillary cartridge (25 capillaries) was run with the following controls: the same brain homogenate as a positive control, extracellular receptor kinase (Erk) as a system control, antibody only, and protein only. The corresponding software, Compass (ProteinSimple®), generated an electropherogram with peaks associated to the expression of proteins of interest and housekeeping proteins, and automatically calculated area under the curve (AUC) for each peak. The high-dynamic range of the exposures (algorithm in software) was used for data analyses in all experiments. To quantify protein levels, the AUC for the GR protein was divided by the AUC for the housekeeping protein (GAPDH). All samples were run as duplicates and randomized across multiple plates; therefore, the ratios were averaged for each animal. All injured animals on a given plate were normalized to shams on the same plate.

### ACTH

Baseline levels of ACTH were measured in rats meeting inclusion criteria. Plasma ACTH levels were quantified using an enzyme-linked immunosorbent assay (ELISA) kit purchased through RayBiotech (Peachtree Corner, GA) (CAT#: EIAR-ACTH-1). ACTH samples were run in duplicate following the manufacturer's instructions. Plasma samples were diluted 50:50 based on manufacturer's recommendation. Mean absorbance for each sample was calculated and the blank optical density was subtracted. The standard curve was plotted using GraphPad software utilizing a four-parameter logistic regression model. Samples were then compared against the standard curve to calculate ACTH levels (pg/mL). Inclusion criteria required that the samples be collected within the first 4 min of the cage being disturbed. The total number of rats differed between the ACTH results and the adrenal glands, as 2 animals exceeded the 4-min cut-off, and 2 animals were outliers (ROUT outlier test, *Q* = 0.1%).

### Histology

Hemispheres from all rats (*n* = 5 group; 20 rats total) were taken after decapitation, drop fixed in 4% paraformaldehyde for 48 h, transferred to fresh PBS with sodium azide, and shipped to Neuroscience Associates Inc. (Knoxville, TN) where they were embedded into two gelatin blocks (MultiBrain® Technology, NeuroScience Associates, Knoxville, TN) to be processed for histological and immunohistochemical staining. Forty-micron thick sections were taken in the coronal plane, stained with ionized calcium binding adaptor molecule (Iba-1); (1° Ab: Abcam, ab178846, 1:14,000; 2° Ab: Vector: BA-1000, 1:1,000); or glial fibrillary acidic protein (GFAP); (1° Ab: Dako, Z0334, 1:75,000; 2° Ab: Vector, BA-1000) using free-floating technique, visualized using 3,3′-Diaminobenzidine (DAB), and wet-mounted on 2%-gelatin-subbed slides. A subset of slides had myelin stained using Weil's method (NeuroScience Associates, Knoxville, TN).

Photomicrographs of the PVN and dentate gyrus (DG) were taken using a Zeiss microscope (Imager A2; Carl Zeiss, Jena, Germany) in bright-field mode with a digital camera using a 40× objective. For the PVN, one digital photomicrograph was acquired per rat across three coronal sections for a total of 3 images per rat. Weil staining of myelin clearly marked the location and morphology of the fornix and optic tract on adjacent sections and was used to confirm location of the PVN. For the DG, 40× images were taken of the superior molecular layer, the polymorph layer, and of the inferior molecular layer (3 sections per rat) for a total of 9 DG images per rat. Representative sections of the DG were chosen to most closely resemble the shape of the DG at −3.12 mm from bregma using the rat brain atlas (62).

### Skeleton Analysis to Quantify Microglia

Microglia were analyzed by an investigator blinded to injury status and sex using computer-aided skeleton analysis as previously published (53, 64). Briefly, photomicrographs were converted to binary images which were skeletonized using ImageJ software (National Institutes of Health, https://imagej.nih.gov/ij/). The Analyze Skeleton Plugin (developed by and maintained here: http://imagej.net/AnalyzeSkeleton) was applied to the skeletonized images, which tags branches and endpoints and provides the total length of branches and total number of endpoints for each photomicrograph. Cell somas were manually counted by two investigators and averaged for each photomicrograph. The total branch length and number of process endpoints were normalized to number of microglia cell somas per image to calculate the number of microglia endpoints per cell and total microglia process length per cell. Data from the three images were averaged to a single representative measure for the PVN. Data from nine images were averaged to a single representative measure for the DG.

### Pixel Density to Quantify Astrocytes

A densitometric quantitative analysis was performed on GFAP tissue staining at 40× magnification using ImageJ software (1.48v, NIH, Bethesda, MD, USA) employing previously published methods by an investigator blinded to injury and sex status (42, 65). Images were converted to binary, the background was subtracted, and each image was digitally thresholded to separate positive-stained pixels from unstained pixels, and then segmented into black and white pixels, indicative of positive and negative staining, respectively. The percentage of GFAP (black) staining was calculated using the following formula: [(Total area measured black/total area measured) × 100 = the percentage of area stained with GFAP]. The percentage of area stained was averaged to a single value representative of each rat for statistical analysis. Stained cells were manually counted for each image using ImageJ's multipoint tool. Only cells with a visible soma were counted. The number of cells in each image was averaged to create a single value for each animal for statistical analysis.

### Statistics

For molecular data, the injured rats were normalized to the same sex sham and an unpaired, two-tailed Student's *t*-test was used to determine whether the change from the same sex sham was statistically significantly different (*p* < 0.05). For all other data, a two-way ANOVA was utilized to test differences as a function of injury and sex followed by a Tukey *post-hoc* analysis. For ACTH, due to variability, the data were log transformed (actual data are shown). Bars represent the mean + standard error of the mean (SEM). ^†^*p* < 0.05 in comparisons to sham (injury effect), ^#^*p* < 0.05 in comparisons to the opposite sex (sex effect), *p > 0.05 in comparison to same-sex sham (*post-hoc*). All statistical data were analyzed via GraphPad Prism software (V8.4.0).

## Results

### Males and Females had Similar Injury Severity, Yet Males Lost More Weight Than Females

**Figure 1:**
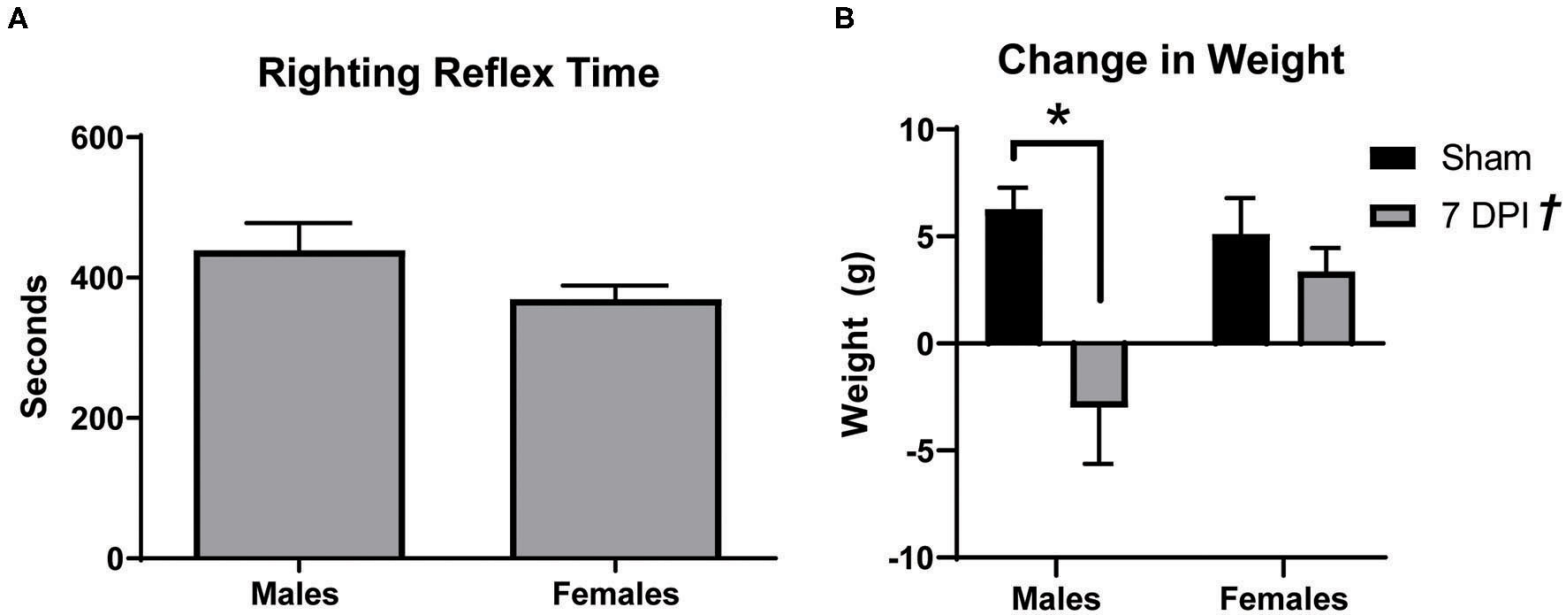
Males lost more weight after diffuse TBI. **(A)** Righting reflex times for male and female rats did not significantly differ (*t*_18_ = 1.685; *p* = 0.1093). **(B)** mFPI had a significant effect on weight [*F*_(1, 37)_ = 20.73; *p* = 0.002], with 7 DPI males losing more weight than their sex-matched sham controls (*p* = 0.0003; male 7 DPI *n* = 10; female 7 DPI *n* = 11). There was also an interaction between injury and sex [*F*_(1, 37)_ = 5.340; *p* = 0.0265], where weight loss at 7 DPI depends on the rat's sex. Data are represented by the mean + SEM; *Difference from same-sex sham; ^†^overall injury effect; male sham *n* = 11; 7 DPI male *n* = 9; female sham *n* = 10; 7 DPI female *n* = 11.

Male Sprague Dawley rats typically lose weight after the first several days following a mFPI with a righting reflex ranging from 6 to 10 min (42). In these experiments, both male and female rats had righting reflex times between 6 and 10 min, respectively, 438.7 ± 39.07 s and 369.0 ± 19.65 s (*t*_18_ = 1.685; *p* = 0.109; Figure 1A). At 7 DPI, male injured rats experienced a weight loss of 3.00 ± 2.63 g compared to a weight gain of 6.27 ± 1.01 g among matched shams. In the female set, there was weight gain in both the injured group (3.36 ± 1.10 g) and matched shams (5.10 ± 1.70 g; Figure 1B). Analysis by two-way ANOVA indicated a statistically significant difference in weight as a function of injury [*F*_(1, 37)_ = 20.73; *p* = 0.002], and *post-hoc* analysis indicated a robust effect in weight change for male injured vs. sham (*p* < 0.001) but not between the female injured and sham rats (*p* = 0.870). Interaction between injury and sex was statistically significant [*F*_(1, 37)_ = 5.340; *p* = 0.027].

### Gene Expression of CRH in the Hypothalamus Did Not Change at 7 DPI

**Figure 2:**
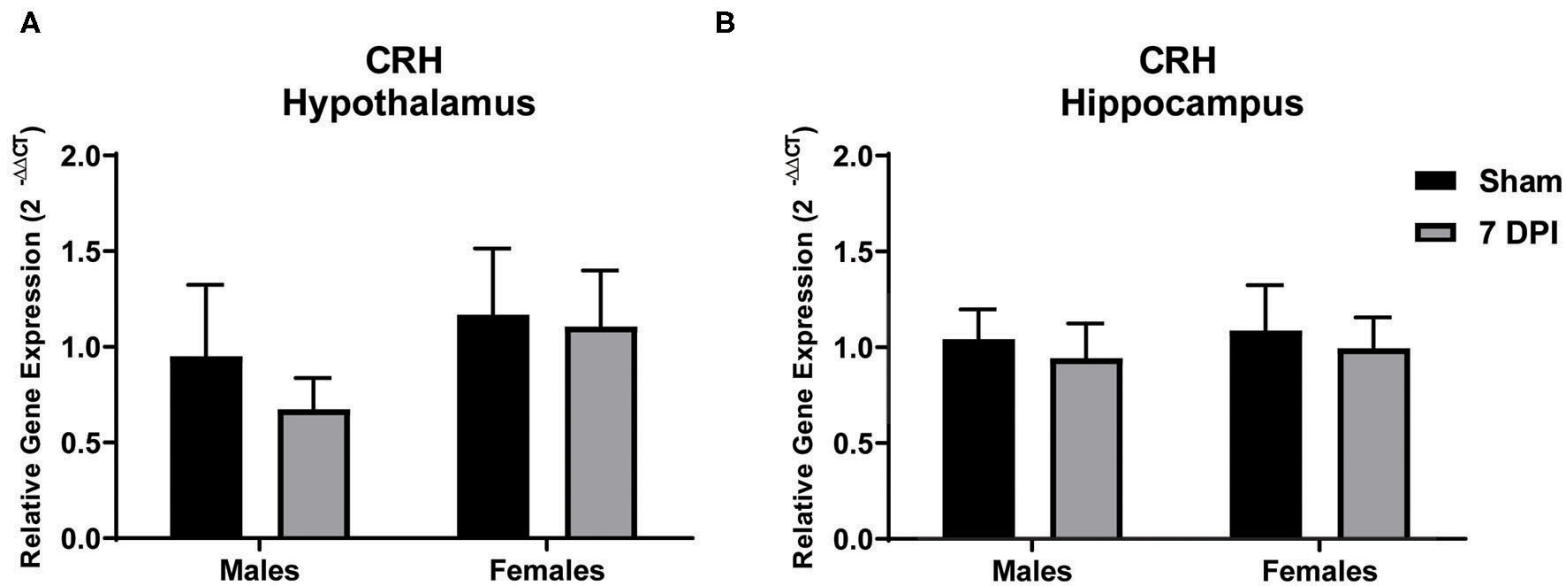
Gene expression of CRH in the hypothalamus did not change at 7 DPI. **(A)** CRH gene expression in the hypothalamus was similar in both males (*t*_9_ = 0.7959; *p* = 0.4466), and females (*t*_10_ = 0.1258; *p* = 0.9024) after injury. **(B)** Results in the hippocampus were similar to hypothalamus, males (*t*_8_ = 0.3830; *p* = 0.7117) and females (*t*_8_ = 0.3348; *p* = 0.7463). Data are represented by the mean + SEM; hippocampus: male sham *n* = 4; 7 DPI male *n* = 6; female sham *n* = 4; 7 DPI female *n* = 6; hypothalamus: male sham *n* = 4; 7 DPI male *n* = 7; female sham *n* = 4; 7 DPI female *n* = 8.

Modulation of the HPA axis is mediated by CRH in the hypothalamus and through feedback mechanisms that include the hippocampus (66). To evaluate CRH gene expression in the sub-acute period following mFPI, mRNA from the hypothalamus and hippocampus of male and female injured and sham rats was evaluated using qPCR. There were no differences in CRH expression in the hypothalamus or hippocampus for male or female injured rats compared to their sex-matched controls: hypothalamus – males (*t*_9_ = 0.796; *p* = 0.447), females (*t*_10_ = 0.126; *p* = 0.902); hippocampus – males (*t*_8_ = 0.383; *p* = 0.712), females (*t*_8_ = 0.335; *p* = 0.746; see Figures 2A,B, respectively).

### ACTH Levels Were Influenced as a Function of Sex at 7 DPI

**Figure 3:**
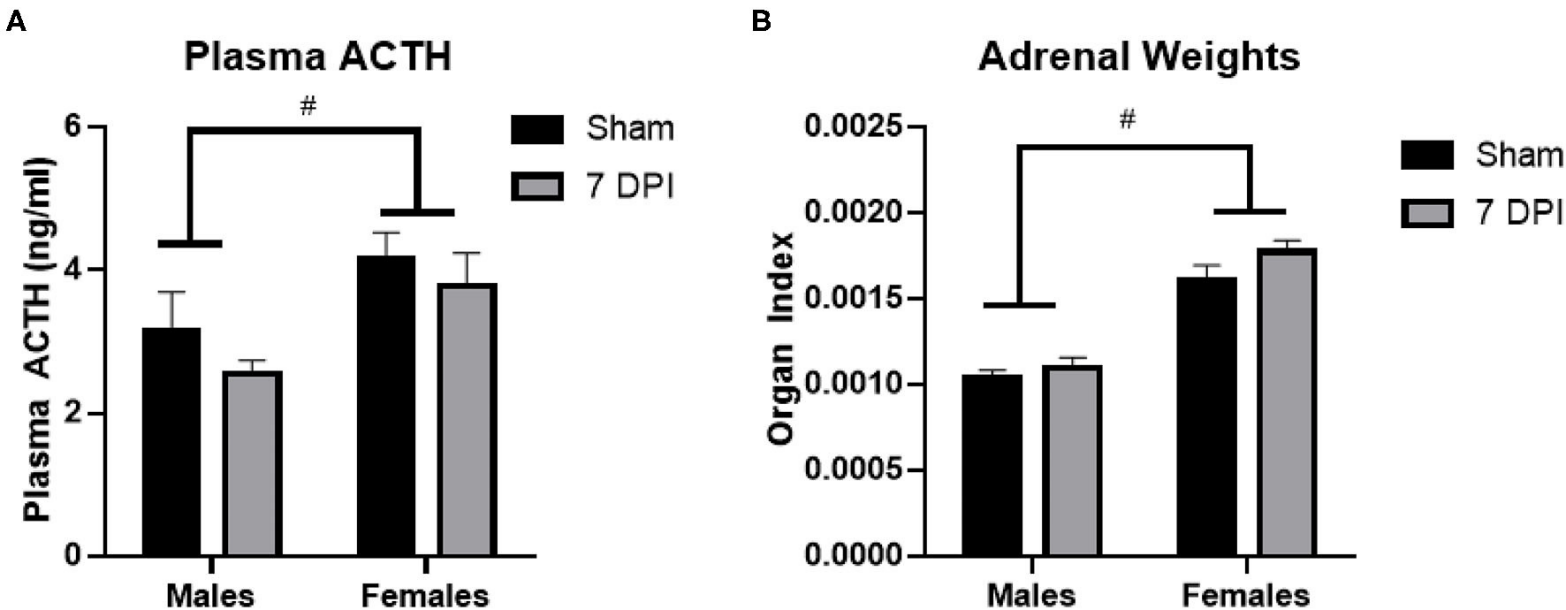
ACTH and adrenal gland weights did not change at 7DPI. **(A)** There was no injury effect on ACTH [*F*_(1, 31)_ = 1.591; *p* = 0.217]. Female rats also had significantly higher ACTH levels compared with males [*F*_(1, 31)_ = 8.742; *p* = 0.006]. **(B)** Adrenal weights were normalized to the body weights of each animal to calculate the organ index. There was no effect of injury [*F*_(1, 37)_ = 2.944; *p* = 0.0946], but the female organ index was significantly higher compared with the organ index of males [*F*_(1, 37)_ = 83.84; *p* < 0.0001]. Data are represented by the mean + SEM. ^#^difference from opposite sex; ACTH: male sham *n* = 8; 7 DPI male *n* = 9; female sham *n* = 8; 7 DPI female *n* = 10. Adrenals: male sham *n* = 11; 7 DPI male *n* = 9; female sham *n* = 10; 7 DPI female *n* = 11.

ACTH is produced in the anterior pituitary and is released into circulation to stimulate the production and release of glucocorticoids from the adrenal glands. Using a two-way ANOVA, ACTH levels were not influenced by injury at 7DPI [*F*_(1, 31)_ = 1.591; *p* = 0.217]. Regardless of injury, ACTH levels overall were higher in females compared to males [*F*_(1, 31)_ = 8.742; *p* = 0.006; Figure 3A].

The weights of the adrenal glands were analyzed as absolute weight and normalized to the rat's body weight, termed an organ index (OI) (67, 68). The absolute weight of the adrenal glands did not differ due to injury between males (0.039 ± 0.001 g) and females (0.041 ± 0.001 g; *p* = 0.106). Analysis of the OI by two-way ANOVA demonstrated no difference at the injury level [*F*_(1, 37)_ = 2.944; *p* = 0.095], but the OI for females was significantly higher compared to males [*F*_(1, 37)_ = 83.84; *p* < 0.0001; Figure 3B]. This difference at the sex-level is due to females having a lower body weight than males.

### Evidence of Microglial Activation in the PVN at 7 DPI in Male Rats

In a sentinel state, microglia are equidistantly distributed with fine ramified processes extended and surveying their local environment. When ramified microglia encounter a stimulus, it can do one or a combination of activate, proliferate, and migrate. *Activated* morphologies have retracted and thickened processes and can initiate either neurotoxic or neurotrophic signaling, depending on the nature of the stimulus with continued signaling through fractalkines, glycoproteins, and cytokines. Amoeboid microglia are fully activated, are indistinguishable from infiltrating macrophages (fully retracted processes), and function to phagocytose cellular debris (69–71). Morphological markers of microglial activation include decreased number of endpoints and shorter branch length and occur alongside the increase in cell numbers, both of which are indicators of a neuroinflammatory response (64, 72). There is clinical evidence that microglia can remain activated at least 17 years after TBI (73), where they are capable of instigating processes that can influence the HPA axis (74, 75).

**Figure 4:**
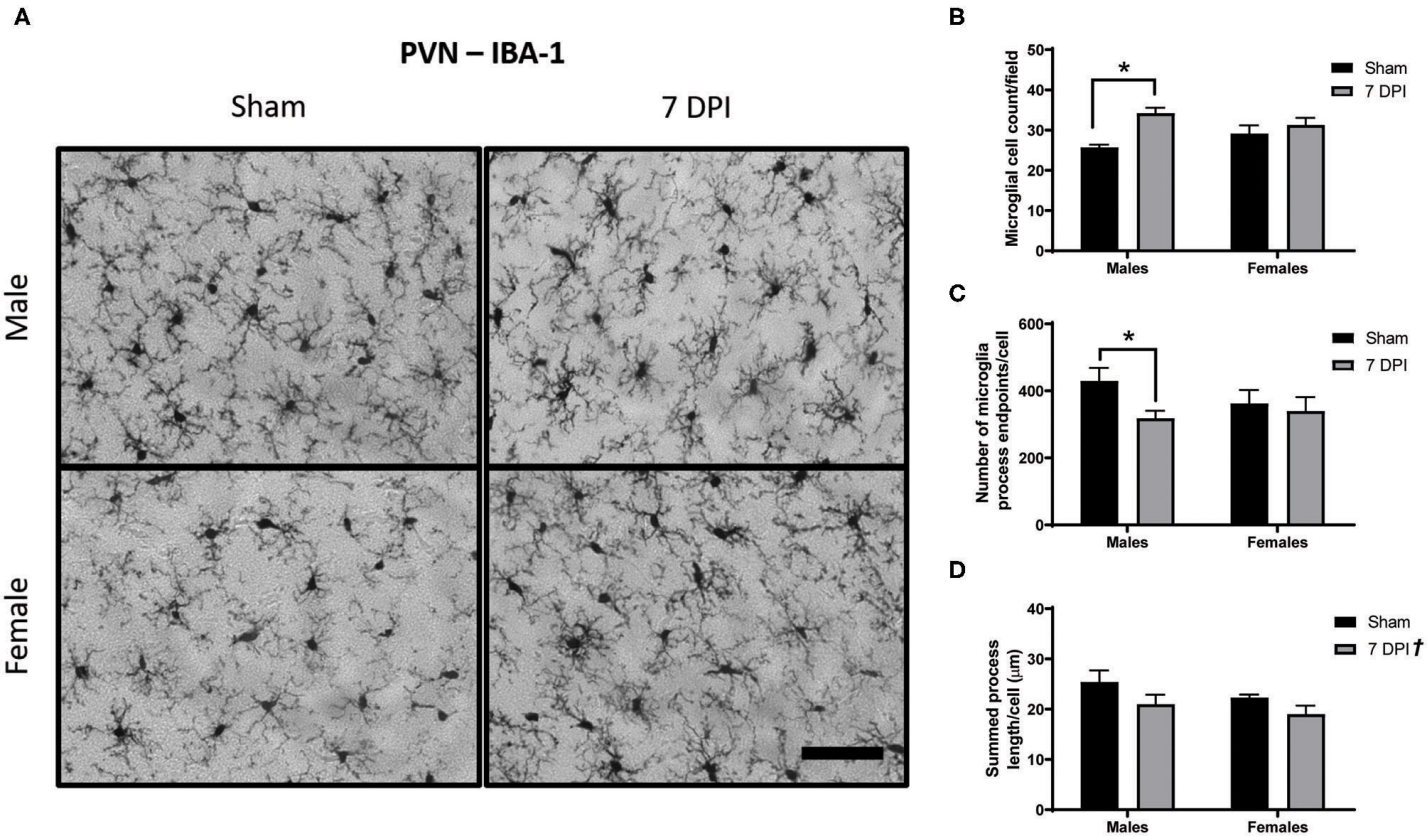
Diffuse TBI activated microglia in the PVN of male rats at 7 DPI. **(A)** 40× representative images of Iba-1 staining in the male (top), female (bottom), sham (left), and 7 DPI (right). **(B–D)** Results from Skeleton Analysis of microglia in the PVN. **(B)** There were significantly more Iba-1 positive cells in males at 7 DPI compared to same-sex shams, but not in females [*F*_(1, 16)_ = 12.32; *p* = 0.0029]. **(C)** The endpoints/microglia approached statistical significance at 7 DPI as a function of injury [*F*_(1, 16)_ = 3.337; *p* = 0.0865]. When stratified by sex and analyzed for an injury effect, there was a statistically significant difference among males (*p* < 0.05) but not among females (*p* = 0.673). There was no overall effect of sex [*F*_(1, 16)_ = 0.3917; *p* = 0.5402]. **(D)** There was an injury effect on the branch length/microglia [*F*_(1, 16)_ = 4.879; *p* = 0.0421] but there were no distinct sex effects [*F*_(1, 16)_ = 2.078; *p* = 0.1688]. Scale bar = 100 μm; data are represented by the mean + SEM; ^†^overall injury effect; *difference from same-sex sham; *n* = 5/group.

Microglial activation after FPI is typically instigated in response to neuropathology. In this model, neuropathology is not prevalent in the PVN (42), but is robust in the DG at 7 DPI (49). Analysis of images from Iba-1 staining in the PVN using Skeleton Analysis (see representative images in Figure 4A) demonstrated a positive association between injury and the number of cells at 7 DPI [*F*_(1, 16)_ = 12.32; *p* = 0.0029]. The difference was notable for male ± 1.30 cells) compared to sex-matched shams (25.74 ± 0.67 cells) [*F*_(1, 16)_ = 12.32; *p* < 0.01], but not among females [(injured) 31.31 ± 1.734 cells vs. (sham) 29.17 ± 2.009 cells; *p* = 0.332]. There was evidence of interaction between injury and sex on the number of cells [*F*_(1, 16)_ = 4.390; *p* = 0.052; Figure 4B], although the statistical significance was marginal.

**Figure 5:**
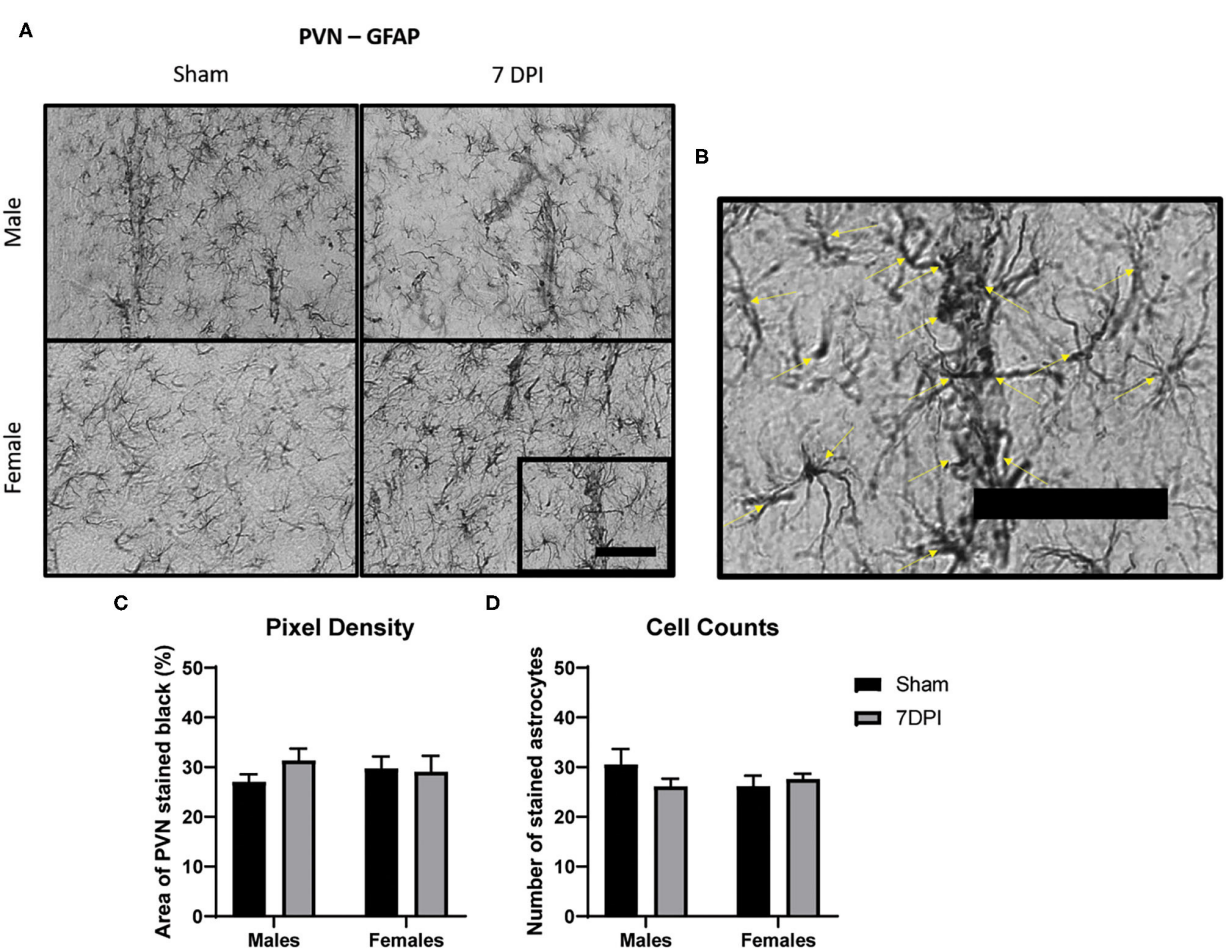
There was no evidence of reactive astrogliosis in the PVN at 7 DPI. **(A)** 40× representative images of GFAP in the male (top) and female (bottom), sham (left), and 7 DPI (right). **(B)** The image within the box in **(A)** is magnified to demonstrate how cell counts were made, where inclusion criteria required the presence of the soma (yellow arrows). **(C)** Pixel density of GFAP staining did not change as a function of injury [*F*_(1, 16)_ = 1.276; *p* = 0.2753] or sex [*F*_(1, 16)_ = 0.4245; *p* = 0.5240]. **(D)** Cell counts of GFAP did not change as a function of injury [*F*_(1, 16)_ = 0.5068; *p* = 0.4868] or sex [*F*_(1, 16)_ = 0.4628; *p* = 0.5061]. Scale bar = 100 μm; data are represented by the mean + SEM; *n* = 5/group.

Injury was associated with fewer endpoints per microglia (Figure 5C). Among males, there were 35% fewer endpoints [(injured) 318.3 ± 22.30 vs. (sham) 429.8 ± 39.04] compared with 6.5% in females [(injured) 340.0 ± 41.13 vs. (sham) 362.2 ± 40.60]. The injury effect in males was statistically significant (*p* < 0.05; Figure 5C); there was no injury effect for females (*p* = 0.673). However, statistical measurement of an injury effect by two-way ANOVA did not reach statistical significance [*F*_(1, 16)_ = 3.337; *p* = 0.087]. There was no effect of sex on number of endpoints per microglia [*F*_(1, 16)_ = 0.392; *p* = 0.540], nor an interaction between injury and sex [*F*_(1, 16)_ = 1.490; *p* = 0.241; Figure 4C].

The average branch length per microglia demonstrated an overall injury effect [*F*_(1, 16)_ = 4.879; *p* = 0.042]; brain injured rats demonstrated shorter process lengths (20.00 ± 1.25) compared with shams (23.86 ± 1.24). There was no effect of sex [*F*_(1, 16)_ = 2.078; *p* = 0.169] and no evidence of interaction [*F*_(1, 16)_ = 0.109; *p* = 0.745; Figure 4D].

### There Was No Evidence of Reactive Astrogliosis in the PVN at 7 DPI

Astrocytes serve in a number of roles in the central nervous system (CNS) including nutrition, metabolism, maintenance of extracellular ion concentrations, active participation in neurotransmission, regulation of cerebral blood flow, and modulation of synaptic plasticity. Astrocyte activation can result from perturbations in homeostasis, immune response, or more invasive tissue destruction caused by trauma. Changes in the intensity of GFAP staining and increased number of cells can provide a useful estimate of the presence and severity of regional disruption.

We analyzed the intensity of GFAP staining in the PVN by quantifying pixel density for dark staining using ImageJ (see representative images at 40× in Figure 5A). Pixel density of GFAP staining did not change as a function of injury [*F*_(1, 16)_ = 1.276; 0.275] or sex [*F*_(1, 16)_ = 0.425; *p* = 0.524; Figure 5C]. Cell counts of GFAP stained astrocytes did not yield differences as a function of injury [*F*_(1, 16)_ = 0.507; *p* = 0.487] or sex [*F*_(1, 16)_ = 0.463; *p* = 0.506; Figure 5D]. Despite evidence of elevated microglia activation in the male PVN at 7 DPI, there was no evidence of reactive astrogliosis in adjacent sections.

### Evidence of Microglial Activation in the DG at 7 DPI in Both Males and Females

**Figure 6:**
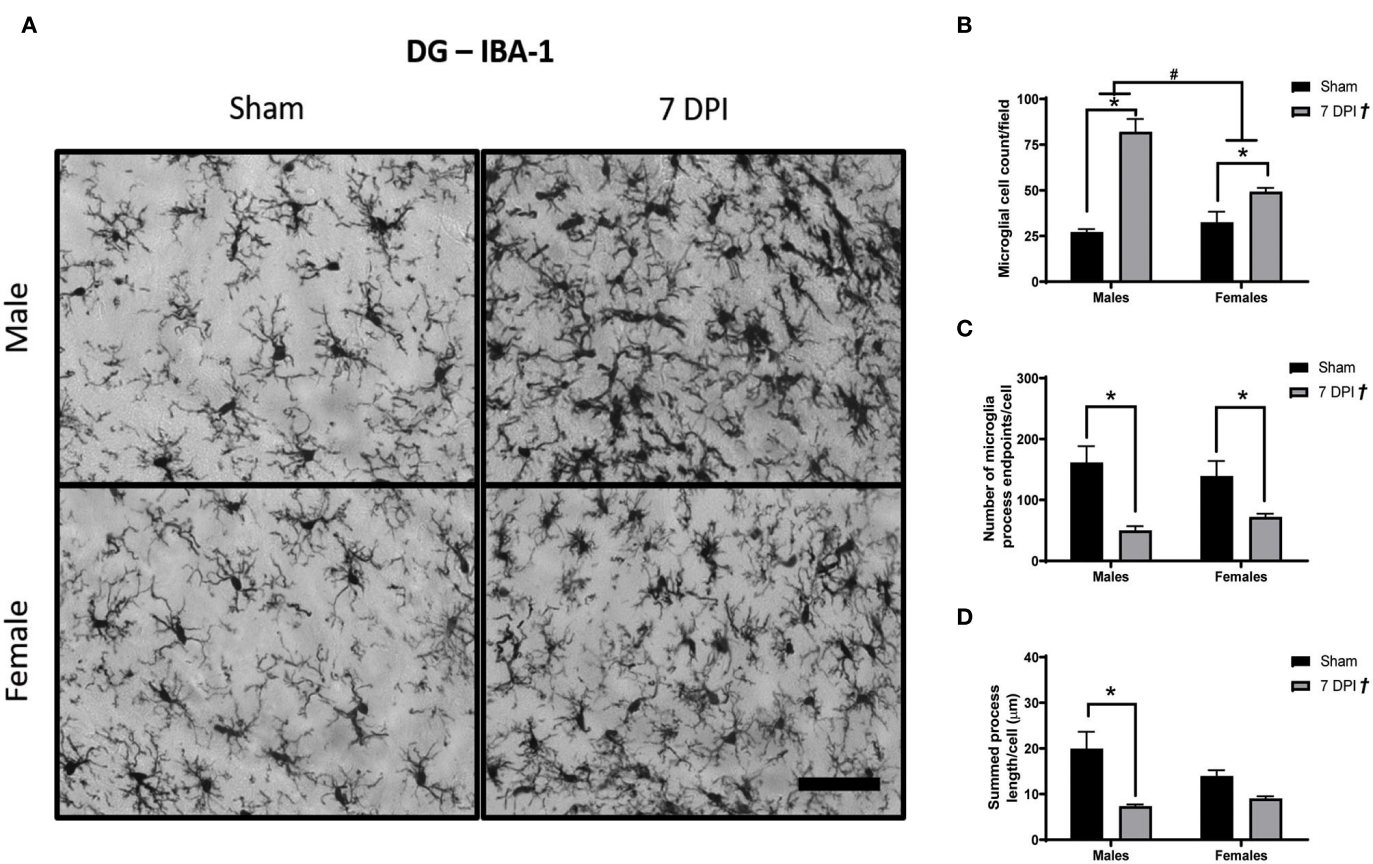
Diffuse TBI activated microglia in the hippocampus across both sexes at 7 DPI. **(A)** 40× representative images of Iba-1 staining in the male (top), female (bottom), sham (left), and 7 DPI (right). **(B)** There was an injury effect on average microglia cell counts at 7 DPI [*F*_(1, 16)_ = 5.58; *p* < 0.0001] in the hippocampus. Both injured males and females had more microglia compared to uninjured shams. Additionally, there was an overall sex effect on number of microglia in the hippocampus [*F*_(1, 16)_ = 8.603; *p* = 0.0097]. There were more microglia in males compared with females. There was an interaction between sex and injury [*F*_(1, 16)_ = 16.60; *p* = 0.0009]. **(C)** There was an overall injury effect on microglia process endpoints per cell [*F*_(1, 16)_ = 22.51; *p* = 0.0002]. endpoints per cell [*F*_(1, 16)_ = 22.51; *p* = 0.0002]. *post-hoc* analysis indicated there were fewer microglia process endpoints per cell at 7 DPI in males (*p* = 0.0034), whereas females approached significance (*p* = 0.095) compared to respective shams in the hippocampus. **(D)** There was an overall injury effect on process branch lengths [*F*_(1, 16)_ = 20.03; *p* = 0.0004] in the hippocampus. *post-hoc* analysis indicated that male injured rats had shorter process branch lengths compared with male shams. Scale bar = 100 μm; data are represented by the mean + SEM; ^†^overall injury effect; ^#^difference from opposite sex; *difference from same-sex sham; *n* = 5/group.

Images of Iba-1 staining in the DG were analyzed using Skeleton Analysis (see representative images in Figure 6A). Microglia cell numbers were significantly higher among injured rats (65.71 ± 6.44 cells) compared to matched shams (29.86 ± 2.94 cells) [*F*_(1, 16)_ = 5.58; *p* < 0.0001; Figure 6B], and the elevated microglial cell counts at 7 DPI (vs. sham) were elevated for both males (*p* < 0.001) and females (*p* < 0.05). The elevated cell counts were more pronounced among male (54.66 ± 9.75 cells) compared to female (40.92 ± 4.01 cells) injured rats [*F*_(1, 16)_ = 8.603; *p* < 0.001; Figure 6B]. There was an interaction between injury and sex [*F*_(1, 16)_ = 16.60; *p* < 0.001].

There were fewer microglia process endpoints per cell as a function of injury at 7 DPI [*F*_(1, 16)_ = 22.51; *p* < 0.001; Figure 6C]. The reduction in endpoints after injury (vs. shams) was statistically significant for males (*p* < 0.01) but not in females (*p* = 0.095), where endpoints decreased in males by 25% and females by 6%. There were also shorter process branch lengths as a function of injury [*F*_(1, 16)_ = 20.03; *p* < 0.001], where *post-hoc* analysis indicated a statistically significant effect in males (*p* < 0.01) but not for females (*p* = 0.318; Figure 6D). A greater number of microglia, fewer endpoints, and shorter branch lengths in the DG represent a robust TBI-induced activation of microglia at 7 DPI.

### Evidence of Astrocytosis in the DG at 7 DPI in Both Males and Females

**Figure 7:**
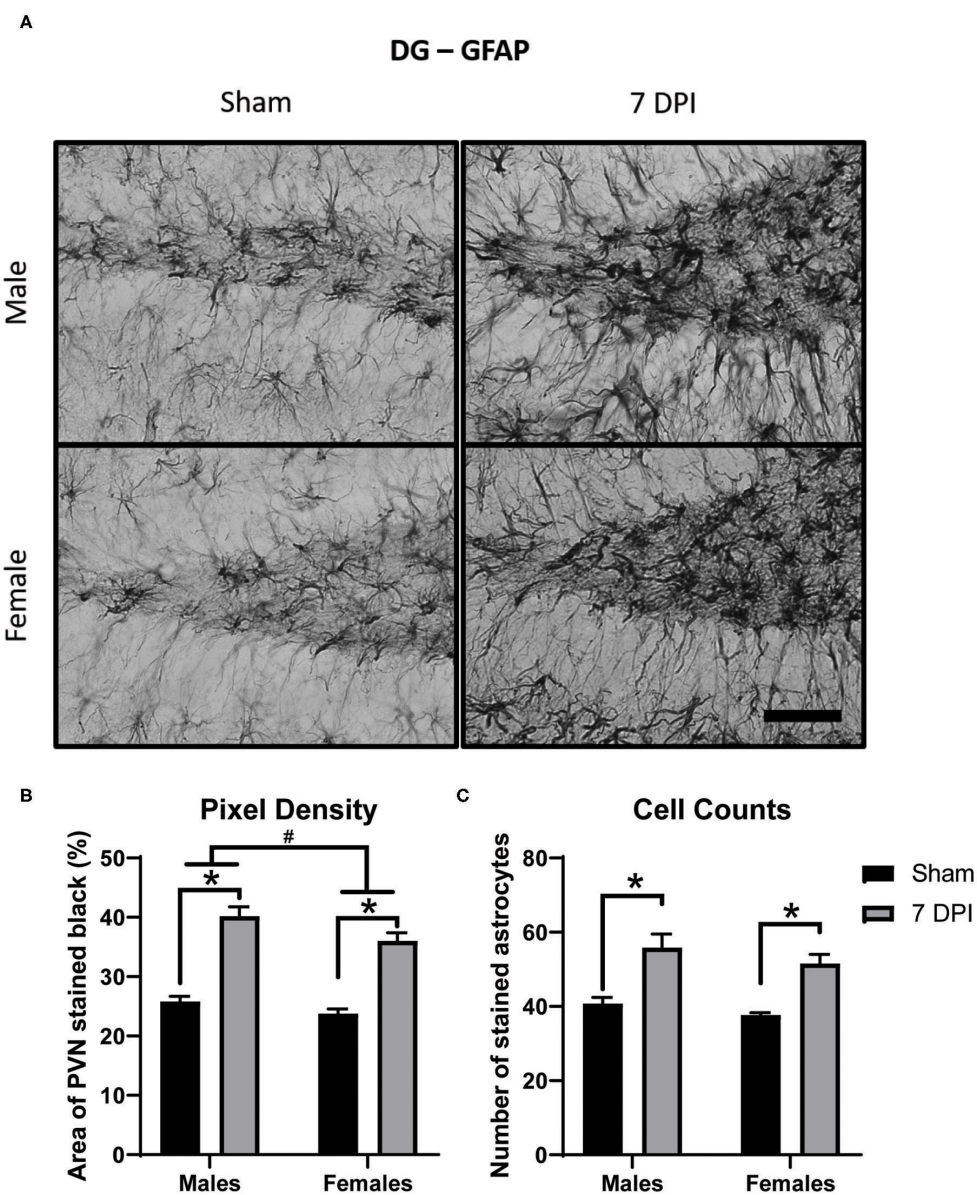
Diffuse TBI led to astrocytosis in the DG across both sexes at 7 DPI. **(A)** 40× representative images of GFAP in the male (top), female (bottom), sham (left), and 7 DPI (right). **(B)** There was an injury effect on pixel density of GFAP [*F*_(1, 16)_ = 124.2; *p* < 0.0001], where both injured males (*p* < 0.0001) and injured females (*p* < 0.0001) had significantly greater in comparison to their sex-matched shams. There was also an effect of sex [*F*_(1, 16)_ = 6.771, *p* = 0.0193], with females having a significantly lower density of GFAP in comparison to males. **(C)** There was an injury effect on cell counts of GFAP stained astrocytes [*F*_(1, 16)_ = 37.25; *p* < 0.0001] but no sex effect [*F*_(1, 16)_ = 2.481; *p* = 0.1348]. Scale bar = 100 μm; data are represented by the mean + SEM; ^#^difference from opposite sex; *difference from same-sex sham; *n* = 5/group.

Astrocytosis is defined as increased intensity of GFAP staining with evidence of increased number of astrocytes. Pixel density of GFAP staining was used to estimate the intensity of GFAP staining in the DG (40× representative images in Figure 7A). There was increased pixel density of GFAP as a function of injury [*F*_(1, 16)_ = 124.2; *p* < 0.001], with greater density at 7 DPI in both male (*p* < 0.001) and female (*p* < 0.001) injured rats compared to matched shams. GFAP differed as a function of sex [*F*_(1, 16)_ = 6.771, *p* = 0.019]; where, independent of injury, females had a lower density of GFAP compared to males (Figure 7B).

There were significantly higher cell counts of GFAP stained astrocytes as a function of injury [*F*_(1, 16)_ = 37.25; *p* < 0.001]; this significant difference was found in both 7 DPI males (*p* = 0.002) and females (*p* < 0.010; Figure 7C). The higher cell counts did not demonstrate an effect by sex [*F*_(1, 16)_ = 2.481; *p* = 0.135], nor an interaction between injury and sex [*F*_(1, 16)_ = 0.064; *p* = 0.803].

### Glucocorticoid Receptor Protein Levels Increased in the Hippocampus of Females at 7 DPI

**Figure 8:**
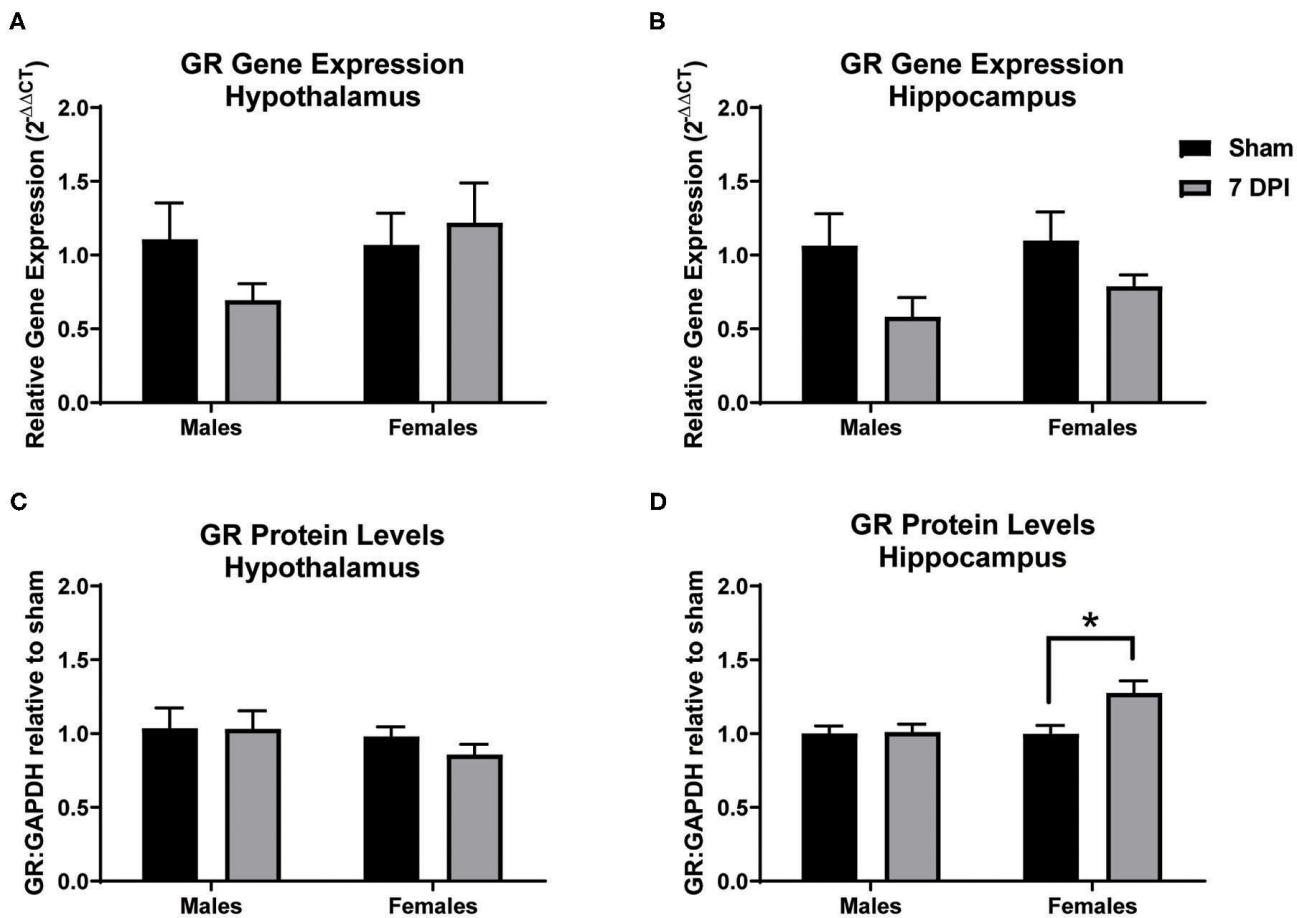
Diffuse TBI led to higher GR protein levels in the hippocampus of females but not males at 7 DPI. **(A)** Gene and protein expression of GR in the hypothalamus did not change as a function of injury in males (*t*_10_ = 1.689; *p* = 0.1220) or females (*t*_10_ = 0.3562; *p* = 7291). **(B)** There were no TBI-induced differences in gene expression of GR in the hippocampus of males (*t*_8_ = 2.042; *p* = 0.0754) or females (*t*_12_ = 1.651; *p* = 0.1246). **(C)** There were no TBI-induced differences in GR protein levels in the hypothalamus in males (*t*_14_ = 0.022134; *p* = 0.9833) or females (*t*_9_ = 1.263; *p* = 0.2365). **(D)** There was a significant injury-induced difference in protein levels of GR in the hippocampus in females (*t*_10_ = 2.797; *p* = 0.0189), but not males (*t*_17_ = 0.1527; *p* = 0.8805). Data are represented by the mean + SEM; *difference from same-sex sham; male sham *n* = 4–5; 7 DPI male *n* = 6–7; female sham *n* = 4–6; 7 DPI female *n* = 8.

Gene and protein levels of glucocorticoid receptors (GRs) were evaluated in the hypothalamus and hippocampus at 7 DPI. There was no difference in the gene expression of GRs in the hypothalamus as a function of injury among males (*t*_10_ = 1.689; *p* = 0.122) or females (*t*_10_ = 0.3562; *p* = 0.729; Figure 8A). Gene expression of GRs did not reach statistical significance in injured males in the hippocampus compared to matched shams (*t*_8_ = 2.042; *p* = 0.075), nor in females (*p* = 0.125; Figure 8B). GR protein levels in the hypothalamus also did not differ at 7 DPI in males (*t*_14_ = 0.022; *p* = 0.983) or females (*t*_12_ = 1.651; *p* = 0.237; Figure 8C). However, the GR protein levels in the hippocampus were 30% higher for females at 7DPI (*t*_10_ = 2.797; *p* = 0.019) but there was no difference in males (*t*_17_ = 0.1527; *p* = 0.881; Figure 8D).

## Discussion

In these experiments, we evaluated mechanisms in the sub-acute time period that may contribute to the development of HPA axis dysregulation by two months post-injury as previously demonstrated in male rats (42). Additionally, we added cycling females to experiments to evaluate sex-differences to further understand the role of sex in chronic symptom presentation following TBI. Our data indicated that weight loss over 7 DPI is profound in males but not for females, with males not rebounding to their sham counterparts weight by 7 DPI; there is no change in CRH gene expression; no change in ACTH levels; increased microglial activation in the PVN in males but not females; increased microglial activation in the DG of both males and females; and coinciding astrocytosis in the DG of both sexes. These data indicated no effect of injury on GR gene and protein levels in the hypothalamus; however, there was evidence of a 30% elevation in protein levels after injury in the hippocampus of females but not males. The results are summarized in Supplementary Figure 4. These data indicate sex-differences in sub-acute pathophysiology following DAI that precede chronic HPA dysregulation. Moreover, these data implicate a potential role for the involvement of gliosis with GRs in instigating chronic HPA axis dysregulation leading to a mild form of adrenal insufficiency.

HPA axis dysregulation and affective symptoms following acquired brain injury, including TBI and stroke, have become increasingly acknowledged in clinical studies (14, 32, 37, 76–80). Despite the prevalence and late-onset nature of affective symptoms after TBI (and stroke), few studies have evaluated the longitudinal pathology and HPA axis regulation for underlying pathology (42, 81–86). Only two studies have included females at the sub-acute timepoint (26, 87) and one study included females at a chronic time point (88). Taylor et al., demonstrated sex-differences using ovariectomized females after controlled cortical impact, where CORT levels were significantly lower in injured males and females compared to sex-matched sham, similar to our previous reports (42, 88). Injured females also demonstrated a lower stress-induced increase in CORT (at 30 min) in comparison to female shams, where males stress-induced CORT levels were similar between sham and injured. These data indicated sex differences in HPA axis regulation after TBI, without the confound of changes in circulating gonadal hormones due to estrous cycles (88). However, the etiology of HPA axis dysregulation following brain injury is poorly understood.

In our study, we found that male and female injured rats had similar righting reflex times. While weight decreased in both sexes over the 7 DPI period, weight loss was significantly greater in injured males compared to sex-matched shams, but this effect was not observed in females. Weight loss after mFPI has been previously reported in male rats (42) but the difference in females was unknown. A decrease in weight is likely due to an observed decrease in food intake for the first 24–48 h post-injury. Sex differences may be due to differential growth rates between male and female rats, the innate differences in mass (females having greater fat mass percentage), the innate ability for females to conserve energy by storing it as fat, and differences in gonadal hormones (89–91). Davis et al., demonstrated that forced fasting for 24 h after focal TBI can cause ketosis in rats which can be neuroprotective, so this weight loss cannot be ruled out as an indication of a natural tendency to promote neuroprotection (92). More studies are needed to explain the significance, if any, of this observation.

Levels of the neuropeptide CRH may play a role in chronic HPA axis dysregulation. CRH is released from the PVN to trigger release of ACTH in the anterior pituitary gland and acts as a central constituent of the HPA axis-mediated stress response. CRH is also produced by interneurons within the pyramidal layers of the hippocampus where CRH levels are thought to increase after severe stress and contribute to the decreased complexity of pyramidal neurons, hippocampus-dependent memory deficits, as well as feedback to the HPA axis (93, 94). CRH mediates pathogenesis in the PVN and amygdala at 2 h following focal TBI in rats (95), yet changes in CRH in the hippocampus at a sub-acute time point post-injury have never been reported. Our results indicated that CRH gene expression was not altered in the hypothalamus or hippocampus of male and female rats at 7 DPI. However, it should be taken into consideration that CRH protein levels could be different, gene and protein levels change as a function of time post-injury, and 7 DPI may not be a time point that demonstrates active changes. Russell et al. recently reported sex-dependent changes in CRH receptor-1 and receptor-2 gene expression, where CRH receptor-2 was sex-dependently altered after blast-induced TBI in the dorsal hippocampus and other limbic regions (87), indicating that while CRH may not change in response to TBI, systemic sensitivity to CRH may be altered. Additional studies are needed to assess possible roles for CRH in the instigation of chronic HPA axis dysregulation.

ACTH is released by the anterior pituitary gland and travels through the blood stream to mediate CORT release from the adrenal cortex. According to previously published methods, using isoflurane for 2 min, does not elevate ACTH or CORT levels in comparison to controls, nor was it found to increase circulating levels of adrenaline or noradrenaline in the plasma (61, 96–98). Baseline plasma ACTH levels were not changed as a function of injury but were higher in females compared to males. Females having a higher level of ACTH has previously been reported and is associated with female HPA axis activation being more robust than in males, reviewed in (99). Since the HPA axis activates very quickly, where ACTH can begin to increase as early as 180 s after the rat is disturbed (96), and blood in these studies was collected between 200 and 240 s, baseline plasma ACTH levels are likely elevated above resting levels. No injury effect on ACTH is in line with no injury effects previously reported for CORT levels in males or females following other forms of experimental TBI at 7 DPI in comparisons between controls (26, 84, 100). These data indicate that changes seen at chronic time points are not present at 7 DPI, and fluctuations in the regulation of the HPA axis may contribute to the development of affective symptoms at later time points.

Adrenal gland weight increases are indicative of chronic stressors and high glucocorticoid production in the rat (101). While there were no significant differences in the actual adrenal weight between males and females, when normalized to the body weight of each individual animal, adrenal glands were proportionately larger in females compared with males, similar to previous reports (102). This is concurrent with other studies that suggest this sex-difference is dependent on the inhibitory effects of testosterone and faciliatory effects of estrogen on the HPA axis (103–106). Further studies examining the morphology of adrenal glands via immunohistochemistry are necessary to establish if the differences are in the zona fasciculata and zona reticularis areas of the adrenal gland as these two areas have been found to have the most sex-differences independent of TBI (107). Russell et al., assessed the adrenal glands for expression levels of 11β-hydroxylase, 11β-hydroxysteroid dehydrogenase 1, and melanocortin 2 receptor in mice after blast injury and found no difference in males or females (26). Together, these data do not indicate changes in the adrenal glands at 7 DPI that would contribute to late-onset chronic HPA axis dysregulation as previously reported in male rats (42).

In the PVN we found a greater number of microglial cells at 7 DPI in males but not in females. There were significantly fewer endpoints per cell in males in comparison to the sex-matched control, but not as part of the 2-way ANOVA. Preliminary data and power calculations were carried out in male rats (not shown), demonstrating similar trends, indicating that this difference may be biologically relevant. A small but statistically significant decrease in branch length was found across both sexes as a function of injury. These data support a neuroinflammatory response in the PVN of male brain injured rats. The increase in cell number in the PVN can result from recruitment and proliferation of resident microglia (108) or the infiltration of peripheral macrophages (109). Activated microglia can play a role in regulation of cytokines, synaptic reorganization, neuron morphology and survival, and glial scarring (110). We previously reported that neuropathology was not apparent at 7 DPI in males (42), indicating that this response is likely mediated by the TBI-induced activation of the HPA axis as reported after stress paradigms (75). Previous publications indicate that activation of microglia in the PVN can be associated with excitation of the sympathetic nervous system and hypertension (111, 112). More studies are required to identify the biological relevance of microglial activation and sex-differences in the pathophysiology and phenotypic longitudinal outcomes.

In the DG, brain injury significantly increased the number of microglia in both males and females in comparison to their sex-matched shams, including an interaction between injury and sex with injured males having a greater number of microglia compared with injured females. Skeletal analysis showed fewer endpoints per cell and shorter branch lengths as a function of injury. These data indicate a robust neuroinflammatory response to DAI in the DG, where the response in males was larger than females, similar to that previously reported after a focal injury (controlled cortical impact) in mice (113), but not after mFPI in mice (114). In our study, we did not follow-up with any behavioral tests to confirm cognitive deficits, but other studies showed that midline FPI-induced age-related microglial activation in the hippocampus was directly related to cognitive decline (115–117).

Gonadal hormone receptors on microglia could also play a role in the lack of neuroinflammatory response in 7 DPI females. Microglia can express several gonadal hormone receptors including androgen receptors (AR), estrogen receptor (ER)-beta, and ER-alpha [although reports are inconsistent; see review (118)], membrane progesterone receptor α (mPRα), and luteinizing hormone receptor (119). ARs were only expressed on activated microglia after neurological insult (120). Activation of ERs and mPRα is thought to dampen the neuroinflammatory response (121–124). Circulating ligands for AR, mPRα, and ERs at the time of tissue extraction may be useful in identifying a role for gonadal hormones in mediating the neuroinflammatory response (125).

In agreement with our previously published data in males, the intensity of GFAP staining in the PVN after injury was similar to sham levels in both males and females (42). Analysis of GFAP stained DG regions showed significant evidence of astrocyte activation in both males and females indicated by both the intensity of staining and the number of cells. There were higher levels of GFAP staining in both males and females post-injury compared to sex-matched shams, however unlike with the Iba-1 stain, there was no interaction between sex and injury despite the fact there was an overall sex-difference with females showing less staining intensity than males. Increased GFAP staining after injury is an indication of activated astrocytes, although it is not a precise indicator of the functions that are being mediated. The presence of activated microglia can mediate astrocyte proliferation to stimulate a more complex immune-inflammatory response, in particular, ramping up cytokine, and adhesion molecules, which are present in the DG, but not the PVN (126). Estrogen can indirectly affect astrocytes to contribute to neuroprotection by enhancing glutamine synthetase that can support glutamate neurotransmission (125). Furthermore, both estrogen and progesterone mediate anti-inflammatory, anti-oxidant, growth factor expression, and glutamate clearance properties that can be neuroprotective in astrocytes and may be linked to lower levels of GFAP staining intensity in sham and injured females compared to males (118, 127).

TBI-induced pathology in the hippocampus was evident by activation of both microglia and astrocytes, which may influence feedback regulation on the HPA axis. No overt pathology in the PVN despite observing microglial activation in males indicates that other factors are in play, potentially including circulating CORT and GR regulation. GR gene expression in the hypothalamus and hippocampus did not change at 7 DPI, similar to what was reported after blast injury in male and female mice (26). Protein levels of GR in the hypothalamus were also similar to sex-matched controls at 7 DPI. However, GR protein levels were increased by 30% in the hippocampus of female rats, not males. GRs have been colocalized to every cell in the CNS, where experimental modulation indicates that GRs can have neuroprotective and neurotoxic attributes by either mobilizing energy to attenuate acute stressors, or, causing glutamate accumulation, respectively (128). GRs can modulate nerve growth factor after brain injury which is thought to help aid in regrowth of neurons but may lead to disrupted circuits (129). GRs in microglia and astrocytes have also been indicated in contributing toward regrowth and repair (130). Chronic dysregulation of the HPA axis can also lead to GR resistance. In these studies, microglia, and astrocytes demonstrate high levels of activation in the hippocampus of female rats, likely due to debris clearing at 7 DPI, yet the role of GRs, especially in regard to gliosis, in these processes is unclear. Further investigation is needed to elucidate the roles of GRs in TBI-induced late-onset HPA axis dysregulation and sex-differences.

Limitations included that the estrous cycle was not tracked in these experiments to correlate outcome measures with circulating gonadal hormones. This is an important consideration, as the extent by which circulating hormones can influence outcome measures are largely unknown. Gene and protein assays were evaluated in the dorsal hippocampus and hypothalamus, while immunohistochemistry was focused on the DG and PVN, therefore more conclusive studies are necessary to evaluate injury and sex effects on CRH and GR levels in the DG and PVN. CORT was not measured in these animals, however, previous publications and unpublished data in another cohort support that CORT levels are not changed at 7 DPI (26, 84, 131).

## Conclusions

In summary, we found injury × sex-dependent weight loss, sex-dependent activation of microglia in the PVN, injury × sex-dependent changes in gliosis in the DG, and a significant increase in GR protein levels in females at 7 DPI. Together, these data indicate sex-differences in the sub-acute pathophysiology following DAI that precede HPA dysregulation. Further understanding of the etiology leading up to late-onset HPA axis dysregulation following DAI could identify targets to stabilize feedback, attenuate symptoms, and improve the efficacy of rehabilitation and overall recovery.

## Data Availability Statement

Our data have been uploaded into a repository. This repository does not have an accession number associated with it, as it takes you directly to the data: https://datadryad.org/stash/dataset/doi:10.5061/dryad.547d7wm63?invitation=Ts60gAyAThinlVjcyrkN0w.

## Ethics Statement

This animal study was reviewed and approved by the Institutional Animal Care and Use Committee (protocol 18–384) at the University of Arizona College of Medicine-Phoenix.

## Author Contributions

CEB, AMC, and TCT wrote the first draft of the manuscript. CEB, AMC, SWR, and TCT processed data, did preliminary analyses, and composed figures. CEB, RKR, and GK performed surgeries and injuries. CEB, GK, TCT, RKR, AMC, and SWR participated in tissue collection. RKR and GK assisted with tissue collection. PG-F provided statistical analyses. TCT conceived the experiments, created the study design, and approved the final version of the manuscript. All authors contributed to writing and editing the manuscript.

## Conflict of Interest

The authors declare that the research was conducted in the absence of any commercial or financial relationships that could be construed as a potential conflict of interest.

## Supplementary Material

Supplemental Figure 1 Hypothalamus dissection biopsy location is highlighted in blue. Coordinate locations are found at top right corner. Image is adapted from (62).

Supplemental Figure 2 Hippocampus dissection biopsy location is highlighted in blue. Coordinate locations are found at top right corner. Image is adapted from (62).

Supplemental Figure 3 Antibodies used in experiments for capillary westerns and immunohistochemistry.

Supplemental Figure 4 Summary of results.
