# Supplementary figures and images for: Sex-Dependent Pathology in the HPA Axis at a Sub-acute Period After Experimental Traumatic Brain Injury
Source: Front Neurol. 2020 Sep 30;11:946. doi: 10.3389/fneur.2020.00946 (PMC7554641; doi:10.3389/fneur.2020.00946)

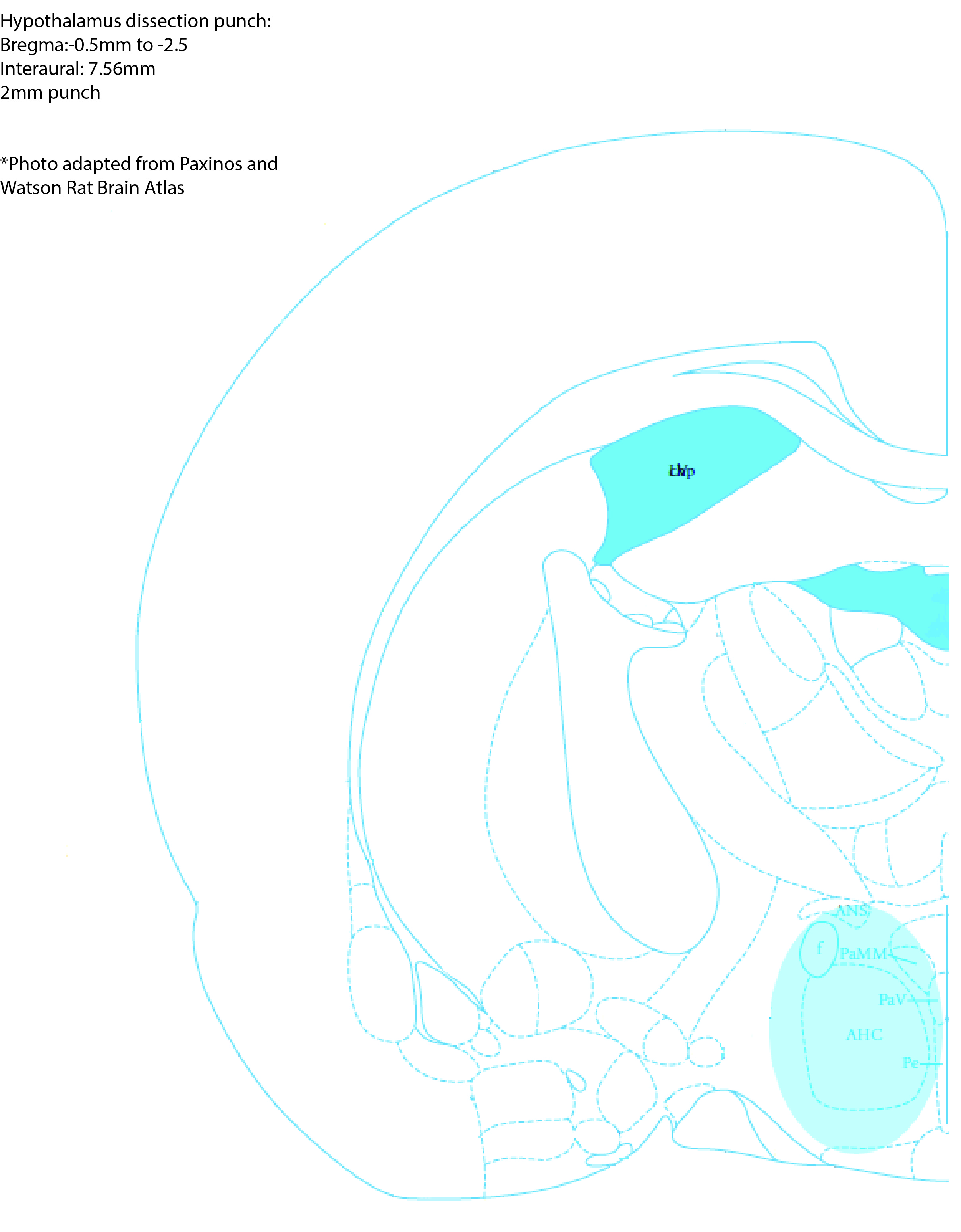

Supplement: Supplemental Figure 1 — Hypothalamus dissection biopsy location is highlighted in blue. Coordinate locations are found at top right corner. Image is adapted from (62). [file Image_1.TIF]

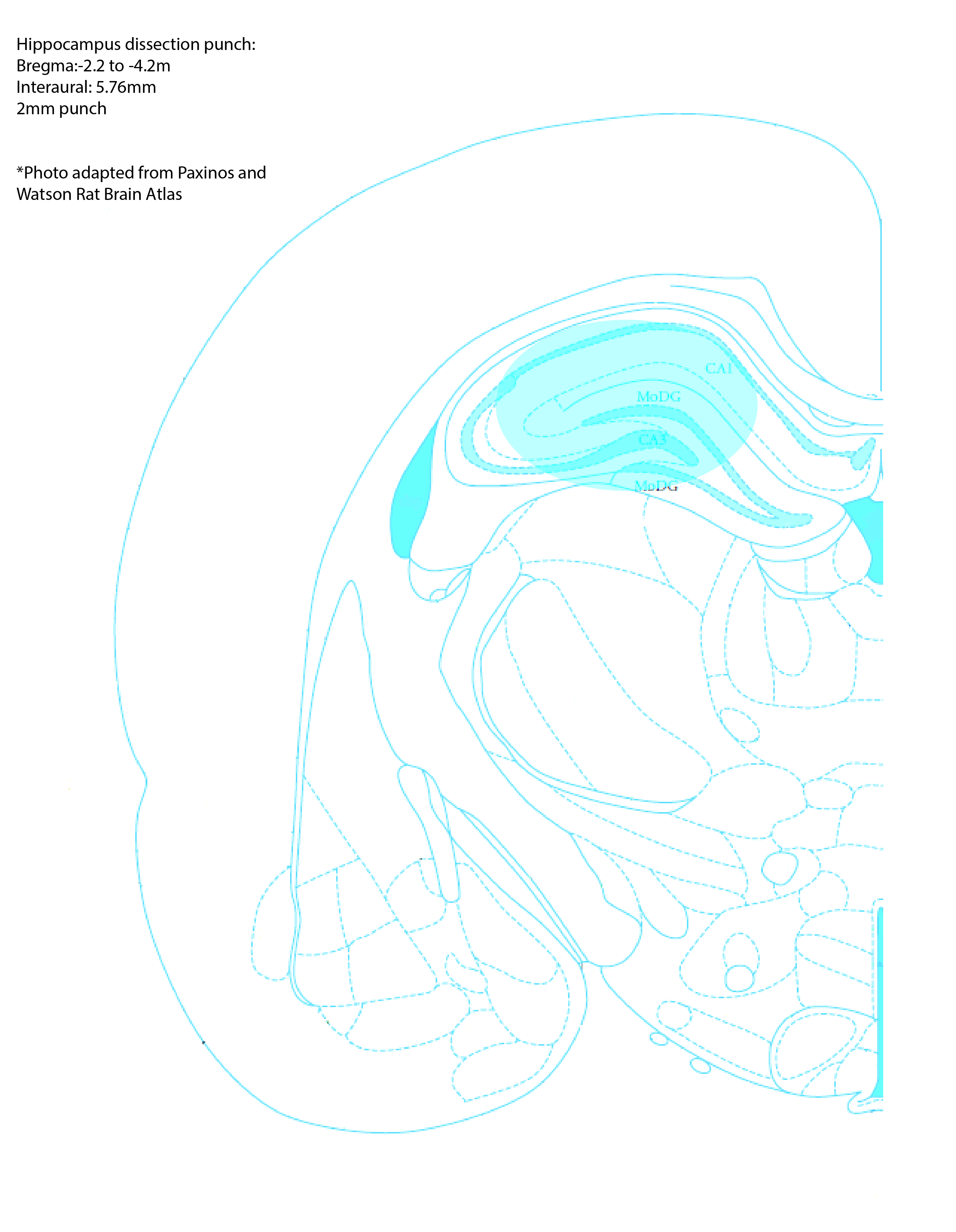

Supplement: Supplemental Figure 2 — Hippocampus dissection biopsy location is highlighted in blue. Coordinate locations are found at top right corner. Image is adapted from (62). [file Image_2.TIF]

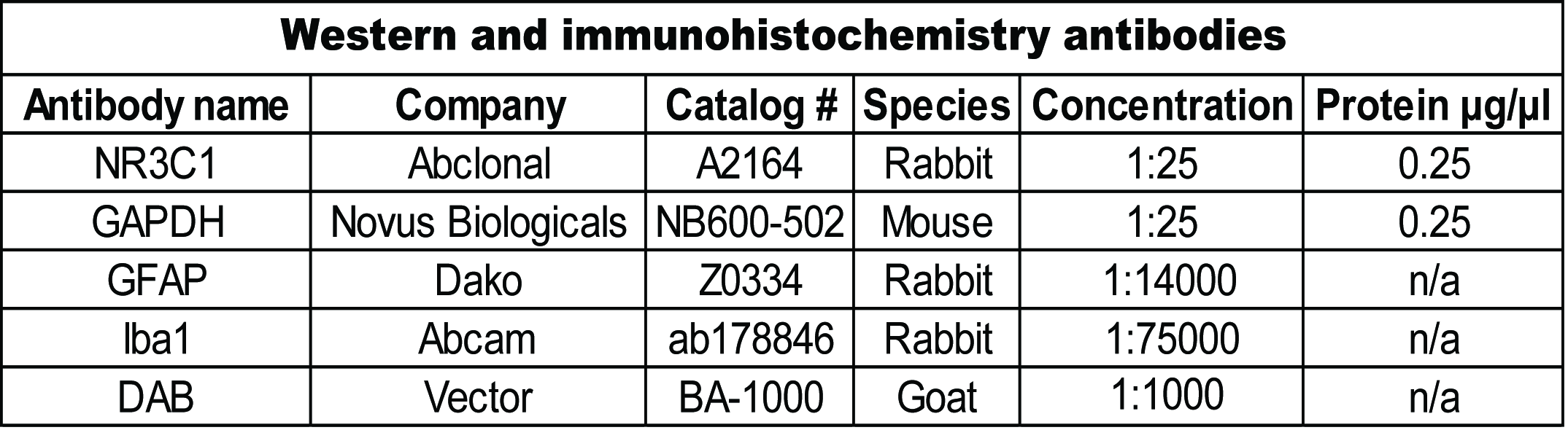

Supplement: Supplemental Figure 3 — Antibodies used in experiments for capillary westerns and immunohistochemistry. [file Image_3.TIF]

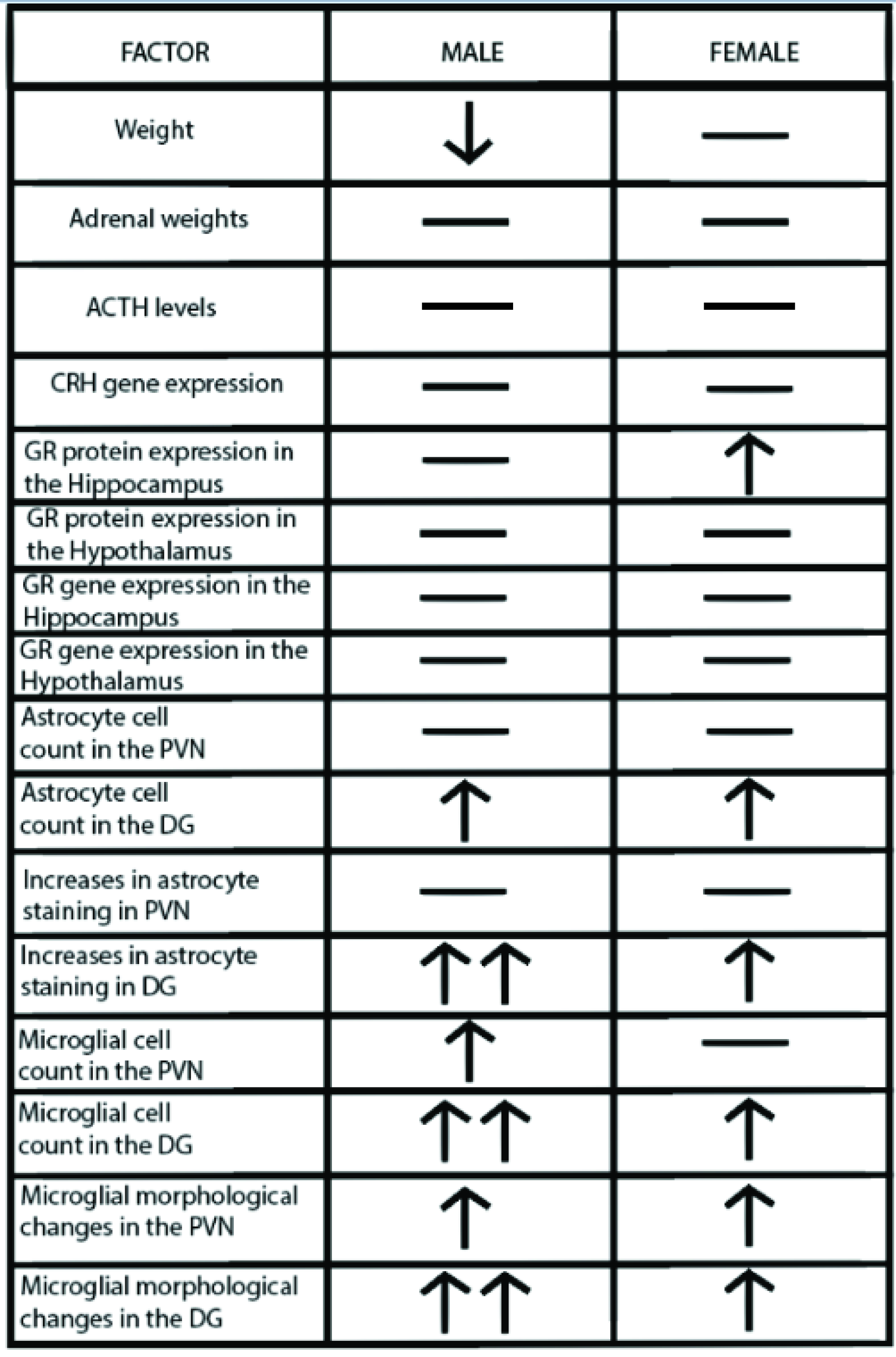

Supplement: Supplemental Figure 4 — Summary of results. [file Image_4.TIF]
